# Effect of milk as a mouthwash on dentin hypersensitivity after non-surgical periodontal treatment

**DOI:** 10.34172/japid.2022.021

**Published:** 2022-11-01

**Authors:** Ashkan Salari, Fereshteh Naser Alavi, Komeil Aliaghazadeh, Masumeh Nikkhah

**Affiliations:** ^1^Dental Sciences Research Center, Department of Periodontics, School of Dentistry, Guilan University of Medical Sciences, Rasht, Iran; ^2^Dental Sciences Research Center, Department of Operative Dentistry, School of Dentistry, Guilan University of Medical Sciences, Rasht, Iran; ^3^Private Practice, Rasht, Iran; ^4^Periodontist, Private Practice, Rasht, Iran

**Keywords:** Allografts, Dentin hypersensitivity, milk proteins, mouthwashes, periodontitis, root planing, scaling

## Abstract

**Background.** Limited evidence is available on the effect of milk as a mouthwash on treating dentin hypersensitivity. The present study aimed to compare the effect of milk as a mouthwash with one anti-hypersensitivity mouthwash in decreasing dentin hypersensitivity after non-surgical periodontal treatment.

**Methods.** Patients with generalized moderate-to-severe chronic periodontitis were selected randomly in the present study and underwent scaling and root planing (SRP). Seventy patients with severe dentin hypersensitivity after two days were assigned to two groups. In group A, the patients were asked to use milk as a mouthwash, and in group B, the patients were asked to use anti-hypersensitiv­ity Misswake mouthwash. The patients’ hypersensitivity was measured during follow-up visits. The independent t-test was used to compare denim hypersensitivity between the two groups. Statistical significance was set at P<0.05.

**Results.** The results showed a significant decrease in dentin hypersensitivity in both groups on days 15 and 30. In the milk group, 11 and 29 patients fully recovered from dentin hypersensitivity on days 15 and 30, respectively. However, in the anti-hypersensitivity mouthwash group, 8 and 27 patients fully recovered from dentin hypersensitivity on days 15 and 30, respectively. Therefore, more patients benefited from the anti-hypersensitivity effects of milk as a mouthwash. However, the differences were not significant during the whole treatment sessions.

**Conclusion.** Using milk as an inexpensive and available mouthwash can decrease dentin hypersensi­tivity after SRP.

## Introduction

 Dentin hypersensitivity is a prevalent clinical problem in dentistry. Dentin hypersensitivity is characterized by short and radiating pain due to chemical, thermal, osmotic, or tactile stimuli that cannot be attributed to any pathological entity.^1^ Dentin is a vital mineralized tissue covered by the enamel in the crown and a thin layer of cementum in the root.^2,3^It contains many tubules that extend from the pulp toward the periphery. Odontoblastic processes are housed within the dentinal tubules and surrounded by the tubular fluid. The hydrodynamic theory is the most widely accepted theory concerning dentin hypersensitivity. This theory attributes dentin hypersensitivity to the fluid movement within the dentinal tubules after chemical, thermal, or osmotic stimulation of the exposed dentin, resulting in the stimulation of delta-A nerve fibers.^4^ There is no dentin hypersensitivity as long as the tissues protecting the dentin, i.e., enamel and cementum, are intact.^5^Many studies have shown a higher prevalence of dentin hypersensitivity in patients with periodontal diseases undergoing non-surgical treatments, especially scaling and root planing.^6^

 Although the severity of dentin hypersensitivity decreases over time, the pain and discomfort resulting from it will adversely affect selecting food, observing proper oral hygiene, and esthetic aspects.^3^ The prevalence of dentin hypersensitivity varies from 1.3% to 68% in different studies. In addition, studies have shown that dentin hypersensitivity can affect all age groups and both genders equally; however, it is more prevalent in female patients in the 30‒40 age group.^7^

 The main factor in dentin hypersensitivity is a defect in the layer protecting the dentin, i.e., enamel and cementum, due to wear lesions, including erosion, abrasion, and attrition, or after gingival recession. However, there is a significant challenge in the diagnosis of other factors involved in the problem, including psychological problems.^8^

 The main factor for treatment success is the correct diagnosis of the etiologic factors for dentin hypersensitivity before undertaking treatment. Unfortunately, despite all the efforts to eliminate the etiologic factors, this condition is still highly prevalent in the community. This strengthens the concept of using anti-hypersensitivity agents necessary to manage this condition. The anti-hypersensitivity agents available on the market contain different agents such as resins, bioactive glass, and milk proteins such as casein phosphopeptides that manage dentin hypersensitivity through various mechanisms, including the occlusion of the dentinal tubules. Different treatments with different mechanisms have been implemented to address this phenomenon; however, none has gained superiority over the others.^8,9^

 It is necessary to evaluate the effects of different mouthwashes on decreasing dentin hyper-sensitivity after non-surgical periodontal treatment in patients with periodontitis since dentin hypersensitivity prevents oral hygiene procedures, resulting in dental plaque and calculus formation and periodontal destruction.^6^ Studies have shown that mouthwashes containing casein phosphoprotein used to treat dental hypersensitivity are expensive and scarce, and it is different for patients to access them.^3,9^ Milk contains casein phosphoprotein, the most inexpensive and available material that can be used at home.

 In the present study, milk was used as a mouthwash containing milk proteins. Only a few studies have evaluated milk as a mouthwash and an anti-hypersensitivity agent to treat dentin hypersensitivity. Therefore, the present study was undertaken to determine the effect of milk mouthwash on treating dentin hypersensitivity after non-surgical periodontal treatment in patients with chronic periodontitis.

## Methods

 In the present interventional study, two groups of patients were selected from those referring to the Department of Periodontics, Faculty of Dentistry. Guilan University of Medical Sciences, using the convenient sampling method based on inclusion criteria. The inclusion criteria consisted of patients in the 18‒60 age range, with no systemic disease, having at least 20 natural teeth, a probing pocket depth of >3 mm, clinical attachment loss of >3 mm in at least 30% of the areas, and severe dentin hypersensitivity (DH=4) after SRP.^10^ In addition, the patients with the following conditions were excluded from the study: a history of diabetes mellitus or other systemic conditions, a history of the use of antibiotics, contraceptives, or anti-inflammatory agents in the past six months, a history of periodontal treatment in the past six months, pregnancy and breastfeeding, smoking and use of alcohol, faulty restoratives, unrestored dental caries or cervical erosion/abrasion and history of anti-sensitivity toothpaste use.

 The sample size was calculated at n=35 in each group at a 5% error coefficient and 80% confidence, considering the odds of dropouts.

 All the patients signed an informed consent form to participate in the study. All the patients had generalized moderate-to-severe chronic periodontitis. The patients were analyzed regarding their brushing technique and the toothpastes they used. If necessary, they were instructed in correct toothbrushing technique with minimal tissue damage, and toothpastes with minimal abrasive power were introduced to the patients. In addition, the patients were asked to refrain from taking sour and acidic foods and fruits.

 A periodontist carried out scaling and root planing (SRP) of the teeth for each patient in one session using an ultrasonic device and manual instruments, followed by polishing in both jaws. To prevent bias, baseline dentin hypersensitivity was determined two days after SRP using the tactile sensation with a #17 dental explorer (JUYA, Pakistan) based on 4VRS (a 4-point verbal rating scale), which is a valuable clinical scale for numerical evaluation of dentin hypersensitivity (Table 1). Only patients with severe dentin hypersensitivity (DH=4) after SRP were included in the study.

**Table 1:** The frequency of dentin hypersensitivity (DH) in follow-up visits in the milk group

**Visits**	**Severe DH** **N (%)**	**Moderate DH** **N (%)**	**Mild DH** **N (%)**	**No DH** **N (%)**
**First visit (Day 4)**	-	21 (60%)	14 (40%)	-
**Second visit (Day 7)**	-	11 (31.4%)	23 (65.7%)	1 (2.9%)
**Third visit (Day 10)**	-	5 (14.3%)	27 (77.1%)	3 (8.6%)
**Fourth visit (Day 15)**	-	1 (2.9%)	23 (65.7%)	11 (31.4%)
**Fifth visit (Day 30)**	-	-	6 (17.1%)	29 (82.9%)

 Seventy patients were randomly assigned to two groups (n=35): A (milk as a mouthwash) and B (an anti-hypersensitivity mouthwash available on the market). The participants in group A used 30 mL of milk (low-fat milk: 1.5%, Kaleh, Iran) at room temperature five times a day for one minute.^10^ The participants in group B used 10 mL of Misswake (Silaneh Sabz Co., Iran) anti-hypersensitivity mouthwash twice daily for one minute. These procedures continued for 30 days.^10^ A researcher, blinded to the sample allocation and the materials used, with adequate training to evaluate dentin hypersensitivity, determined dentin hypersensitivity severity during the recall visits (on days 4, 7, 10, 15, and 30) with a dental explorer using the tactile sensation. The data were analyzed with SPSS 22. Means and standard deviations were used for descriptive statistics. The independent t-test was used to compare quantitative variables between the two groups. Statistical significance was set at P<0.05.

## Result

 In the present study, there were 14 male and 21 female patients with a mean age of 39.71±11.03 years in group A; there were 20 male and 15 female patients with a mean age of 39.11±10.58 years in group B. Table 1 shows the severity of dentin hypersensitivity during the follow-up sessions after SRP in group A. In the milk mouthwash group, during the first visit (day 4), 21 patients had moderate dentin hypersensitivity (60%), and 14 had mild dentin hypersensitivity (40%), with none exhibiting severe dentin hypersensitivity. During the last visit (day 30), none of the patients had severe or mild dentin hypersensitivity, while six patients exhibited mild dentin hypersensitivity (17.1%), and 29 had no hypersensitivity (82.9%). These findings indicate a significant decrease in dentin hypersensitivity on days 15 and 30 in the milk mouthwash group.

Table 2 shows dentin hypersensitivity during the follow-up sessions after SRP in group B. In the Misswake mouthwash group, in the first visit (day 4), three patients exhibited severe dentin hypersensitivity (8.6%), which is different from the milk mouthwash group; 18 patients had moderate dentin hypersensitivity (51.4%), and 14 had mild hypersensitivity (40%). In the last visit (day 30), none of the patients had severe or moderate hypersensitivity, while 8 had mild dentin hypersensitivity (22.9%), and 27 had no dentin hypersensitivity (77.1%). Therefore, there was a significant decrease in dentin hypersensitivity on days 15 and 30.

**Table 2:** The frequency of dentin hypersensitivity (DH) in follow-up visits in the mouthwash group

**Visits**	**Severe DH** **N (%)**	**Moderate DH** **N (%)**	**Mild DH** **N (%)**	**No DH** **N (%)**
**First visit (Day 4)**	3 (8.6%)	18 (51.4%)	14 (40%)	-
**Second visit (Day 7)**	-	15 (42.9%)	18 (51.4%)	2 (5.7%)
**Third visit (Day 10)**	-	6 (17.1%)	27 (77.1%)	2 (5.7%)
**Fourth visit (Day 15)**	-	1 (2.9%)	26 (74.3%)	8 (22.9%)
**Fifth visit (Day 30)**	-	-	8 (22.9%)	27 (77.1%)

Table 3 compares dentin hypersensitivity in all the sessions after SRP in both groups. The results showed a significant decrease in dentin hypersensitivity on days 15 and 30 in both groups. The dentin hypersensitivity after SRP in the milk mouthwash group after 30 days was less severe than that in the Misswake mouthwash group; however, the difference was not significant. In group B, in the first visit, three patients had severe dentin hypersensitivity; in group A, none of the patients had severe dentin hypersensitivity. However, the difference was not significant. In addition, the decrease in dentin hypersensitivity was not significantly different in all the recall visits in the two groups.

**Table 3:** Comparison of the frequency of dentin hypersensitivity (DH) in follow-up visits between the two groups

**Visits**	** Group milk** **DH (mean ± SD)**	**Group mouth wash** **DH (mean ± SD)**	**P-value**
**First visit (Day 4)**	2.6±0.49	2.68±0.63	0.2
**Second visit (Day 7)**	2.28±0.51	2.37±0.59	0.13
**Third visit (Day 10)**	2.05±0.48	2.11±0.47	0.7
**Fourth visit (Day 15)**	1.71±0.51	1.8±0.47	0.2
**Fifth visit (Day 30)**	1.17±0.38	1.22±0.42	0.23

## Discussion

 A prevalent clinical problem after non-surgical periodontal problems is dentin hypersensitivity. The SRP removes the thin cervical cementum, making the tooth sensitive to thermal or environmental stimuli, which decreases the patient’s quality of life and interferes with oral hygiene procedures. Therefore, the dental plaque and calculus are formed again, resulting in continued clinical attachment loss and progression of chronic periodontitis.^11^ The present study evaluated the effects of milk as a mouthwash and an anti-hypersensitivity mouthwash on treating dentin hypersensitivity after SRP in patients with chronic periodontitis. The patients were evaluated for a month because, in many cases, dentin hypersensitivity resolves slowly after SRP and might take a few weeks and even last three months.^1^ The results showed the good efficacy of Misswake anti-hypersensitivity mouthwash in significantly decreasing dentin hypersensitivity; these changes were significant 15 and 30 days after using the mouthwash. Misswake anti-hypersensitivity mouthwash contains sodium fluoride (220 ppm) and tripotassium citrate, which are also important agents to relieve dentin hypersensitivity. Sodium fluoride occludes the dentinal tubules by forming fluoride crystalline salts and precipitating them on the surface of the exposed dentin, relieving dentin hypersensitivity. The mechanism of action of tricalcium citrate is through penetration into exposed dentinal tubules toward the pulp and affecting the nerves to relieve pain.

 Another finding of the present study was the positive effect of milk mouthwash on decreasing dentin hypersensitivity of patients with chronic periodontitis, which was similar to the effect of Misswake anti-hypersensitivity mouthwash. In this line, after 15 days, only one patient had moderate dentin hypersensitivity, and after 30 days, most patients had no dentin hypersensitivity. Madhurkar et al^10^ showed the positive effect of milk mouthwash and sentosil-F anti-hypersensitivity mouthwash on decreasing dentin hypersensitivity, consistent with the present study. Sabir et al^6^ evaluated the effect of milk and warm water as mouthwashes on decreasing hypersensitivity resulting from non-surgical periodontal treatment. Milk mouthwash decreased dentin hypersensitivity after SRP, consistent with the present study. In addition, Madhavan et al reported that agents containing the milk protein CPP-ACP decreased dentin hypersensitivity after SRP.^12^ Milk is an emulsion and colloidal structure with a pH value of 6.7. Casein phosphopeptide (CPP) has a phosphorylated serine and glutamic amino acid sequence as an important dentin anti-hypersensitivity agent. CPP binds to free calcium and phosphate ions through the phosphorylated serine and glutamic amino acid in its structure to form CPP-ACP. In the oral environment, ACP is insoluble, and binding to CPP prevents its precipitation, creating a super-saturated state of calcium and phosphate ions next to teeth.^6,10^ When the oral environment becomes acidic, casein is precipitated, and calcium and phosphate ions are released. Therefore, these ions reach porous lesions and dentinal tubules through the CPP binding to teeth, resulting in the re-formation of apatite crystals, which is the mechanism of dentin desensitization.^2,10^ In the present study, using milk as a mouthwash in patients with chronic periodontitis resulted in the formation of CPP-ACP on tooth surfaces, decreasing dentin hypersensitivity. There is no concern about milk carcinogenicity. Recent studies have reported that milk consumption is associated with the lowest caries experience or incidence. Reasons for these favorable caries-related properties include the lower acidogenicity of lactose and the protective effects of calcium, phosphate, proteins, and fats.^10,14,15^

 Given the relatively high cost of anti-hypersensitivity mouthwashes and the difficulty of obtaining them by patients and the availability and low cost of milk, it can easily be used at home, with the least side effects, to exert its positive effects, as a good alternative for chemical products on the market.

## Conclusion

 Considering the limited number of patients included in the present study, it can be concluded that using milk and Misswake as mouthwashes resulted in decreased dentin hypersensitivity and its relief in patients with chronic periodontitis after SRP. Milk as a mouthwash exhibited efficacy as an anti-hypersensitivity agent similar to Misswake anti-hypersensitivity mouthwash. Therefore, using milk as a mouthwash and desensitizing agent can significantly decrease dentin hypersensitivity after non-surgical periodontal treatment, increasing the quality of life in chronic periodontitis patients.

## Acknowledgments

 None.

## Competing interests

 The authors declare that they have no competing interests concerning the authorship and/or publication of this paper.

## Authors’ contributions

 AS prepared the proposal. AS and FN set and entered the results of the studies and interpreted them, prepared the final report and results, and wrote the manuscript. AS supervised the design and execution of the study and prepared the final report. KA contributed to the preparation of the proposal, conducted the research, and collected the data. All authors approved the final manuscript.

## Funding

 None.

## Availability of data

 The raw data from the reported study are available upon request from the corresponding author.

## Ethics approval

 The study protocol was approved by the Ethics Committee of Guilan University of Medical Sciences under the code IR.GUMS.REC.1398.164.
